# Weber B Distal Fibular Fracture Diagnosed by Point-of-care Ultrasound

**DOI:** 10.5811/cpcem.2016.11.32270

**Published:** 2016-12-07

**Authors:** James Makinen, Jessica Koehler, Sam Tirgari, David Amponsah

**Affiliations:** * Wayne State University School of Medicine, Department of Emergency Medicine, Detroit, Michigan; † Henry Ford Health System, Department of Emergency Medicine, Detroit, Michigan

## Abstract

We report the case of a 45-year-old woman who presented to the emergency department (ED) after an acute ankle inversion injury. After history and physical exam suggested a potential fracture, point-of-care ultrasound (POCUS) demonstrated a cortical defect of the distal fibula, consistent with fracture. Plain radiography failed to demonstrate a fracture. Later, the fracture was identified as a Weber B distal fibular fracture by stress-view radiography. This case reviews the evaluation of acute ankle injuries in the ED and the utility of POCUS as a supplemental imaging modality in the evaluation of ankle fracture.

## INTRODUCTION

Ankle injuries are a common presenting complaint in the emergency department (ED), comprising approximately 5% of all ED visits.^1^ It is important to accurately differentiate ankle fractures and other serious injuries requiring orthopedic consultation from less serious injuries that may be managed conservatively. The current standard of care for the evaluation and diagnosis of ankle fracture, when indicated by the Ottawa Ankle Rules (OARs), is plain film radiography. The OARs are designed to decrease radiographic evaluation of ankle injuries; they have a high sensitivity but very low specificity for fracture diagnosis. This results in many false positives with increased radiation exposure and additional costs. Additionally, plain film radiography is not completely sensitive for ankle fracture, resulting in false negatives. Furthermore, radiographic imaging necessitates radiation exposure and additional cost. This report describes the value of point-of-care ultrasound (POCUS) as a supplemental imaging modality in the evaluation of ankle injuries to aid in making the diagnosis of ankle fracture.

## CASE REPORT

**Image 1:**
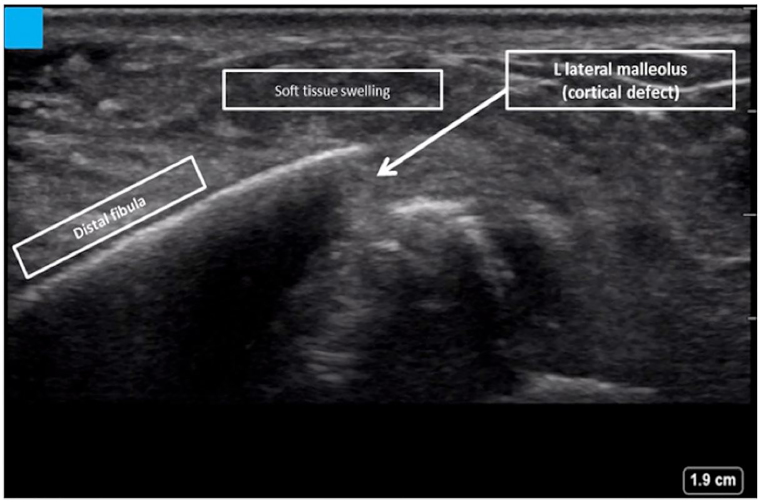
Longitudinal point-of-care ultrasound image of the distal fibula demonstrating a cortical defect.

**Image 2:**
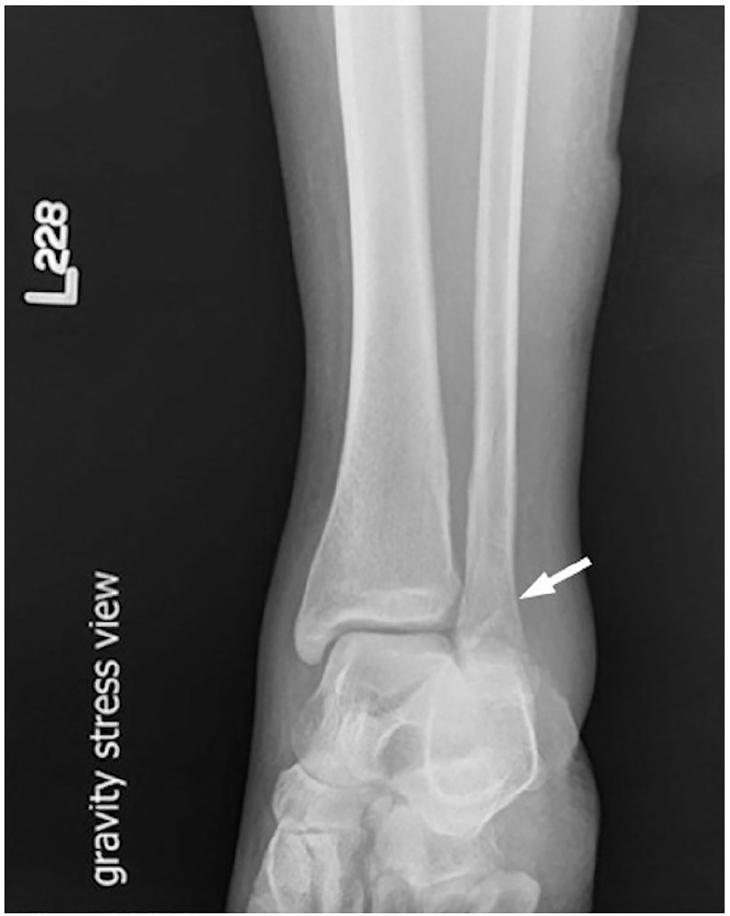
Stress view radiography of the ankle demonstrating an irregularity of the lateral malleolus cortex consistent with a non-displaced Weber B fracture.

A 45-year-old previously healthy woman presented to the ED with a chief complaint of left ankle pain after a fall while roller skating, which resulted in an inversion injury to the left ankle. The patient presented approximately one hour after the injury. She reported no history of surgery or trauma to the ankle prior to the presenting injury. On physical examination, she was in mild distress with stable vital signs. Examination of the left ankle revealed intact skin and moderate soft tissue swelling. There was tenderness to palpation over the distal aspect of the left lateral malleolus and the calcaneofibular and anterior talofibular ligaments. There was mild tenderness over the medial malleolus, and no tenderness to the base of the fifth metatarsal. Passive range of motion was limited due to pain, and the patient could not bear weight on the left foot. Sensation was intact over the foot and ankle, and the pedal pulses were intact. The remainder of the physical exam was unremarkable. POCUS was performed in the ED. Longitudinal views of the left distal fibula demonstrated an obvious cortical defect of the left lateral malleolus consistent with fracture (Image 1). Plain radiographs of the left ankle, foot and lower leg were then obtained, which failed to demonstrate evidence of left fibular fracture. Due to the US findings suggestive of fracture, stress view radiographs of the left foot, ankle and leg were obtained with orthopedic consultation. These views demonstrated a non-displaced Weber B fracture of the left fibula (Image 2). Given this diagnosis, the patient was managed with a medical walking boot, acetaminophen/codeine for pain management and outpatient orthopedic follow up in 2–3 days. At follow up, orthopedics recommended that she continue use of the walking boot for 4–6 weeks.

## DISCUSSION

While ankle injuries are a common presenting complaint in the ED, only 15% of undifferentiated patients presenting with ankle injury can be expected to have evidence of ankle fracture on plain radiographs.^1^ Therefore, efforts have been made in recent years to rule-out ankle fracture by means other than radiography, most notably through the use of the OARs. These clinical decision rules have a high sensitivity, but a very low specificity, resulting in a large number of normal radiographs despite positive OAR findings.^1^ However, the OARs have been independently validated as aiding emergency physicians in a faster diagnosis with fewer radiographs and decreased time in the ED for patients.^2^

When suggested by history and physical exam, the diagnosis of ankle fracture is most commonly made by plain radiography. However, plain radiography has an estimated sensitivity of 85.2% for lateral complex ankle fractures, suggesting a significant percentage of ankle fractures may go undiagnosed.^3^ Additionally, 2014 meta-analysis suggested that after normal plain radiography the occult fracture rate may be as high as 24%.^4^ Therefore, more advanced imaging, such as stress view radiography, computed tomography (CT) or magnetic resonance imaging (MRI), are warranted when the patient’s history and physical exam are strongly suggestive of ankle fracture, even if initial plain radiography is normal.

As demonstrated in this case, POCUS may be used as an extension of the physical exam and provide critical information to ensure a diagnosis of fracture is not missed, especially when plain radiography is normal. Previous studies have analyzed cases in which ankle fracture was falsely ruled out by clinical exam or plain radiography but detected by POCUS, with detected occult fracture rates ranging from 8.96–14.1%.^3^,^5^ Studies have estimated US to have a sensitivity of 87.3–100% and specificity of 93.1–99.1% for the evaluation of ankle fracture. 3,6,7 By one study, sensitivity is estimated to be 96.4% when radiography and US are used in combination to evaluate for ankle fracture, demonstrating the utility of US as a supplemental imaging modality.^3^ US has additional utility in the pediatric population, where radiation exposure is especially concerning. The positive and negative likelihood ratios of US to diagnose ankle fracture in the pediatric population has been estimated to be between 9–20 and 0.04–0.08, respectively.^3^ However, US is notoriously user-dependent, and generalizations about its diagnostic accuracy are difficult to make. Nevertheless, in the hands of a skilled user, US can provide important information in the evaluation of ankle fracture.

The Danis-Weber system is commonly used to characterize ankle fractures. This patient’s fracture was consistent with a stable Type B fracture, which is defined as a spiral fracture of the lateral malleolus of the distal fibula, beginning at the level of the joint and including a partial syndesmotic injury.^8^ Type B fractures may be associated with a stable ankle joint. However, in the setting of a ruptured deltoid ligament, a Type B fracture is unstable.^9^ Ankle stability is commonly determined using stress view radiography, where distal joint space clearing of 2mm or greater is indicative of an unstable joint.^8^

As in this case, ED management for a patient with a stable Type B fracture should include immobilization with a medical walking boot, weight bearing as tolerated, adequate pain control and referral to an orthopedic surgeon for surgical evaluation and follow up. Stress view radiography should be obtained to determine ankle stability, as unstable Type B fractures frequently require surgical fixation.

A missed or mischaracterized ankle fracture may have consequences on the future health of the patient. Inadequate immobilization may result in further damage, putting the patient at greater risk for the development of post-traumatic osteoarthritis, which accounts for 70% of all cases of ankle osteoarthritis.^10^ POCUS can play a critical role in identifying and correctly characterizing ankle fractures. POCUS is an important imaging modality in fracture assessment due to its availability at the patient’s bedside, ease of use, and multiplanar diagnostic capabilities. Its usefulness includes injury assessment for fracture when radiographs are not immediately available, and detecting occult fractures not revealed on radiographs. Sonographic evaluation of bone, however, has limitations and should always be coupled with radiographs and possibly advanced imaging modalities such as CT and MRI when clinically indicated.

## References

[1] Heyworth J (2003). Ottawa ankle rules for the injured ankle. Br J Sports Med.

[2] Perry J, Stiell I (2006). Impact of clinical decision rules on clinical care of traumatic injuries to the foot and ankle, knee, cervical spine, and head. Injury.

[3] Najaf-Zadeh A, Nectoux E, Dubos F (2014). Prevalence and clinical significance of occult fractures in children with radiograph-negative acute ankle injury. Acta Orthopaedica.

[4] Wang C, Shieh J, Wang T (1999). Sonographic detection of occult fractures in the foot and ankle. J Clin Ultrasound.

[5] Singh A, Malpass T, Walker G (1990). Ultrasonic assessment of injuries to the lateral complex of the ankle. Arch Emerg Med.

[6] Ekinci S, Polat O, Günalp M (2013). The accuracy of ultrasound evaluation in foot and ankle trauma. Am J Emerg Med.

[7] Atilla O, Yesilaras M, Kilic T (2014). The accuracy of bedside ultrasonography as a diagnostic tool for fractures in the ankle and foot. Acad Emerg Med.

[8] Singh R, Kamal T, Roulohamin N (2014). Ankle fractures: a literature review of current treatment methods. Open Journal of Orthopedics.

[9] Martin A (2004). Weber B Ankle Fracture: An unnecessary fracture clinic burden. Injury.

[10] Mehta S, Rees K, Cutler L (2014). Understanding risks and complications in the management of ankle fractures. Indian J Orthop.

